# Changes in Pancreatic Fat Content Following Diet-Induced Weight Loss

**DOI:** 10.3390/nu11040912

**Published:** 2019-04-23

**Authors:** Yixin Jiang, Manuela Spurny, Ruth Schübel, Tobias Nonnenmacher, Christopher L. Schlett, Oyunbileg von Stackelberg, Cornelia M. Ulrich, Rudolf Kaaks, Hans-Ulrich Kauczor, Tilman Kühn, Johanna Nattenmüller

**Affiliations:** 1 Heidelberg University Hospital, Diagnostic and Interventional Radiology, Im Neuenheimer Feld 110, 69120 Heidelberg, Germany; Yixin.Jiang@med.uni-heidelberg.de (Y.J.); Manuela.Spurny@med.uni-heidelberg.de (M.S.); ruth.schuebel@gmx.de (R.S.); tno144@googlemail.com (T.N.); christopher.schlett@uniklinik-freiburg.de (C.L.S.); oyunbileg.stackelberg@med.uni-heidelberg.de (O.v.S.); Hans-Ulrich.Kauczor@med.uni-heidelberg.de (H.-U.K.); 2 German Cancer Research Center (DKFZ), Division of Cancer Epidemiology, Im Neuenheimer Feld 581, 69120 Heidelberg, Germany; r.kaaks@Dkfz-Heidelberg.de (R.K.); t.kuehn@Dkfz-Heidelberg.de (T.K.); 3 Department of Diagnostic and Interventional Radiology, Medical Center, Faculty of Medicine, University of Freiburg, 79106 Freiburg, Germany; 4 Huntsman Cancer Institute and Department of Population Health Sciences, University of Utah, 2000 Circle of Hope, Salt Lake City, UT 84112-5550, USA; neli@hci.utah.edu

**Keywords:** magnetic resonance imaging, pancreatic fat content, insulin sensitivity, ectopic fat accumulation, diet-induced weight loss

## Abstract

Background: Obesity can lead to ectopic pancreatic fat accumulation and increase the risk for type 2 diabetes. Smaller intervention trials have shown a decrease in pancreatic fat content (PFC) with weight loss, and we intended to investigate the effects of weight loss on PFC in a larger trial. Methods: Data from the HELENA-Trial, a randomized controlled trial (RCT) among 137 non-diabetic obese adults were used. The study cohort was classified into 4 quartiles based on weight change between baseline and 12 weeks post-intervention. Changes in PFC (baseline, 12 weeks and 50 weeks post-intervention) upon weight loss were analyzed by linear mixed models. Spearman’s coefficients were used to obtain correlations between anthropometric parameters, blood biochemical markers, and PFC. Results: At baseline, PFC only showed a significant correlation with visceral adipose tissue (VAT) (*r* = 0.41). Relative changes in PFC were significantly (*p* = 0.01) greater in Q4 (−30.8 ± 5.7%) than in Q1 (1.3 ± 6.7%). These differences remained similar after one year. However, when adjusting the statistical analyses for changes in VAT, the differences in PFC between Q1 and Q4 were no longer statistically significant. Conclusion: Weight loss is associated with a decrease in PFC. However, the reduction of PFC is not independent from reductions in VAT. Unlike VAT, PFC was not associated with metabolic biomarkers.

## 1. Introduction

Obesity, defined as a BMI above 30 kg/m^2^, is a major risk factor for various chronic diseases, such as diabetes, cardiovascular diseases, musculoskeletal disorders, and several types of cancer [1]. The increasing incidence of obesity worldwide is considered a global pandemic and public health issue [2]. Obesity is related to ectopic fat accumulation in organs, such as the heart, muscle, liver, and pancreas [3], which is also a risk factor for metabolic syndrome. Pancreatic fat infiltration in and around islets could be associated with impaired beta cell dysfunction [4,5,6]. The consequences of pancreatic fat deposition have been poorly analyzed compared to those of non-alcohol fatty liver disease [7]. However, there are some studies indicating that pancreatic steatosis in obesity may increase the risk of pancreatitis, metabolic syndrome, and type 2 diabetes mellitus (T2DM) [8,9]. A positive relationship between pancreatic fat deposition and beta cell dysfunction has been reported in normoglycemia, prediabetes and diabetes [5,6,10].

Weight loss plays a central role in the management of overweight patients with metabolic syndrome [1], and several studies have been conducted to investigate the effects of weight loss on pancreatic fat content (PFC). Tene et al. [11] and Gaborit et al. [12] reported a decrease in PFC after bariatric surgery or exercise-induced weight loss. While Steven et al. [13] and Vogt et al. [14] found no PFC reduction after surgery or calorie restriction-induced weight loss. Thus, the effect of weight loss on PFC still remains unclear. Moreover, it remains largely unknown whether PFC is an independent determinant of metabolic health with distinct pathophysiological consequences or rather a correlate of visceral obesity. In this context, Heber et al. [15] found that visceral adipose tissue (VAT) was the only independent predictor of PFC.

The main aim of this study is to investigate the effect of moderate diet-induced weight loss on PFC among non-diabetic overweight and obese individuals. We further evaluated whether changes in PFC upon weight loss were independent from changes in visceral adipose tissue volume (VAT) and whether PFC was related to a distinct profile of the circulating biomarkers of glucose metabolism, lipid metabolism, adipokine signaling, and inflammation.

To this end, we used data on overweight and obese non-diabetic individuals who participated in a dietary intervention trial with a 12-week intervention and a subsequent follow-up phase and a final assessment 12 months after the baseline measurement, including MR imaging with MRI-derived proton density fat fraction (PDFF) mapping at three time points, to analyze PFC.

## 2. Materials and Methods

### 2.1. Study Population

Data from the HELENA-Trial (trial registration number: NCT02449148 ClinicalTrials.gov), a randomized dietary intervention study that was performed to investigate the metabolic effects of intermittent vs. continuous calorie restriction at the German Cancer Research Centre (DKFZ), Heidelberg, and Heidelberg University Hospital [16], was used in a post-hoc manner to investigate the effects of overall weight loss on PFC. Before enrolment, the study was approved by the ethics committee of the medical faculty of the University of Heidelberg, Germany. Briefly, 150 non-diabetic obese non-smokers (50% females) aged 35–65 years without severe chronic diseases (kidney or hepatic dysfunction, major cardiovascular diseases, history of cancer); without diagnosis of diabetes mellitus; and without HbA1c levels ≥6.5% and/or fasting plasma glucose levels >126 mg/dL measured at screening took part in the study between May 2015 and May 2017. Given these exclusion criteria, the study cohort consisted of metabolically healthy, non-diabetic overweight or obese adults. When applying the criteria of the American Heart Association (AHA) for metabolic syndrome (fasting glucose >100 mg/dL; waist circumference >88 cm for women and >102 cm for men; blood pressure >130/85 mmHG; triglycerides >150 mg/dL or HDL <40 mg/dL in men and <50 in women), only 16% of the study population showed metabolic syndrome, i.e., at least three of the above-mentioned criteria [17]. Participants were randomly assigned to three groups (intermittent calorie restriction (ICR), continuous calorie restriction (CCR), control group) for a 12-week intervention phase, a 12-week maintenance phase, and a 26-week follow-up phase. Written informed consent was obtained from all participants prior to the start of the study. All individuals received biweekly phone calls from dietitians during the intervention phase to assess possible side effects and to monitor self-reported compliance. The analytical sample for the present study consisted of 137 out of 150 initial study participants (6 individuals did not complete the intervention phase (2 unknown reasons, 1 personal/stress, 2 long hospital stay, 1 pregnancy), and PFC values were not available for another 7 because of missing MRI data).

A detailed characterization of the study participants (including questionnaire assessments, medical examinations, blood draws, and MRI imaging) was performed at baseline, after the intervention phase of 12 weeks, and after the follow-up phase of 50 weeks.

The method and study design of the HELENA trial is described in detail in two previous publications [16,18]. The main results with regard to the pre-specified primary and secondary outcomes have recently been published [18].

Side effects of calorie restriction interventions: There were no reports of major adverse effects [18]. Minor physical symptoms including dizziness, tiredness, lack of concentration, and feeling cold were reported by ten members in the ICR group, two in the CCR group, and none in the control group [18]. Adverse effects were only seen in the intervention phase and not in the maintenance or follow-up phases [18]. For further details, see previous publication Schübel et al. [18]

### 2.2. Laboratory Methods

Clinical biochemical indexes (ALT, AST, GGT, HDL, total cholesterol, triglycerides, fasting glucose, HbA1c) were measured by routine assays after blood drawing at the Central Laboratory, Heidelberg University Hospital. Remaining samples were processed and frozen at −80 °C. Plasma levels of adiponectin, leptin, resistin, insulin, C-reactive protein (CRP), tumor necrosis factor-α (TNF-α), interleukin-6 (IL-6), and interferon-γ (IFN-γ) were measured by electrochemiluminescence immunoassays (ECLIA) on a Quickplex SQ 120 instrument from Meso Scale Discoveries (MSD, Maryland, USA) using singleplex and multiplex kits from MSD in the of Division Cancer Epidemiology at the German Cancer Research Centre (DKFZ), Heidelberg. Further details about the biospecimen collection have been published in more detail elsewhere [18].

### 2.3. Imaging

A 1.5 Tesla MR scanner (MAGNETOM Aera; Siemens Healthcare, Erlangen, Germany) was used to measure pancreatic fat content (PFC), liver fat content (LFC), abdominal visceral adipose tissue (VAT), and subcutaneous adipose tissue (SAT). Hardware, MR protocol, and software remained the same among all MR scans in this study.

**Figure 1:**
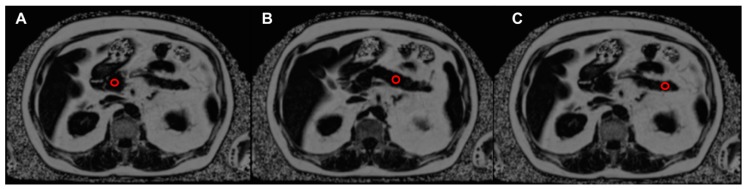
Assessment of pancreatic fat content on a proton density fat fraction (PDFF) map with the position of three regions of interest (ROIs, red circles). (**A**) Pancreatic head; (**B**) pancreatic body; (**C**) pancreatic tail.

Pancreatic fat content was quantified by a multi-echo GRE technique (Siemens LiverLab, Siemens Healthcare, Erlangen, Germany; see Figure 1) [16,19]. Regions of interest (0.785 cm^2^) were manually placed in the pancreas head, body, and tail on the proton density fat fraction map (PDFF) using a post-processing software (OsiriX, Pixmeo SARL, Bernex, Switzerland; see Figure 1). The regions of interest were positioned by avoiding artefacts, vessels, possible focal lesions, and adjacent visceral fat. The measurement of the three regions of interest was taken constantly for all three time points (Baseline, week 12, and week 50) of each participant. Intra- and inter-reader variability was assessed in 40 cases. Intra- and inter-reader reproducibility was high (ICC = 0.968, 0.953). The reliability of PDFF measurements has been validated previously [6]. Liver fat content (LFC) was measured in three regions of interest (4 cm^2^ each). These were placed dorsally, anterior-medially, and anterior-laterally of the right liver lobe in a slice slightly cranial of the liver hilum on a PDFF map using the same post-processing software (OsiriX, Pixmeo SARL, Bernex, Switzerland). Details of the measurement of liver fat content have been described previously [20,21].

The quantified measurement of SAT and VAT was performed by a 2-point DIXON sequence from the neck to the upper legs. An algorithm developed in-house and based the Medical Imaging Interaction Toolkit (MITK) software was used to semi-automatically measure SAT and VAT volume [22].

### 2.4. Statistical Analyses

Main findings from the HELENA trial related to the question of whether intermittent calorie restriction is superior to continuous calorie restriction regarding body composition, adipose tissue gene expression, and metabolic parameters have been published recently, without statistically significant differences between intermittent and continuous calorie restriction [18]. For this project, the study cohort was re-classified for post-hoc analysis into quartiles based on change in body weight between baseline and 12 weeks post-intervention, irrespective of the dietary method used to achieve this weight loss. Quartile cut-off points were as follows: Quartile 1 (≤1.9% weight loss, *n =* 33), Quartile 2 (>1.9% to ≤4.5% weight loss, *n =* 35), Quartile 3 (>4.5% to ≤7.5% weight loss, *n =* 35), and Quartile 4 (>7.5% weight loss, *n =* 34). Given the post hoc character of the present study, these quartile cut-off points were based on the distribution of weight loss values after the intervention. Quartiles, rather than a priori defined cut-off points, were used to achieve the best possible statistical power, with equally large groups. In the context of this post-hoc categorization, it is important to note that intermittent vs. continuous calorie restriction did not have differential effects on PFC and other body composition parameters, which facilitated the present analyses on overall weight loss (irrespective of the dietary method used to achieve it) and PFC. Linear mixed models with age and sex adjustment were used to analyze the effect of weight loss on changes in PFC. We used log-relative differences to assess changes in body composition over time, as for a small minority of participants, body composition or anthropometric parameters showed slight increases, and potential inequalities due to scaling in this situation could be avoided by log-transformation. Spearman’s coefficients were used to assess the correlations between anthropometric parameters, blood biochemistry markers (ALT, AST, GGT, HDL, total cholesterol, triglycerides, fasting glucose, HbA1c) and pancreatic fat content, but also VAT and liver fat content. SAS 9.4 (Cary, NC, USA) was used for statistical analyses.

## 3. Results

### 3.1. Characteristics of the Study Population at Baseline

Baseline characteristics of the study population are shown in Table 1. PFC values at baseline were 10.3 ± 5.1%, 7.7 ± 4.1%, 10.1 ± 11.2%, and 8.6 ± 4.2% from Q 1 to Q 4. The mean age of individuals in Q4 (47.4 ± 8.3) was slightly lower than in the other quartiles (Q1, Q2, and Q3: 51.0 ± 6.3, 51.2 ± 8.3, and 51.2 ± 8.3). Weight, BMI, waist circumference, and VAT levels at baseline were highly similar across the four groups.

### 3.2. Characteristics of Pancreatic Fat with Blood Biomarkers and Body Fat Volumes

Correlations of pancreatic fat content, liver fat content, and VAT with BMI, weight, waist circumference, blood pressure, VAT, SAT, LFC, and blood biomarkers are shown in Table 2. The only parameter for which we observed a correlation of rho > 0.4 with pancreatic fat content was VAT (rho = 0.41, *p* < 0.001). There were no correlations at rho > 0.4 between PFC and anthropometric measurements or blood biomarkers. By contrast, VAT and LFC showed stronger correlations with some of the biomarkers.

### 3.3. Effects of Weight Loss on Pancreatic Fat

**Figure 2:**
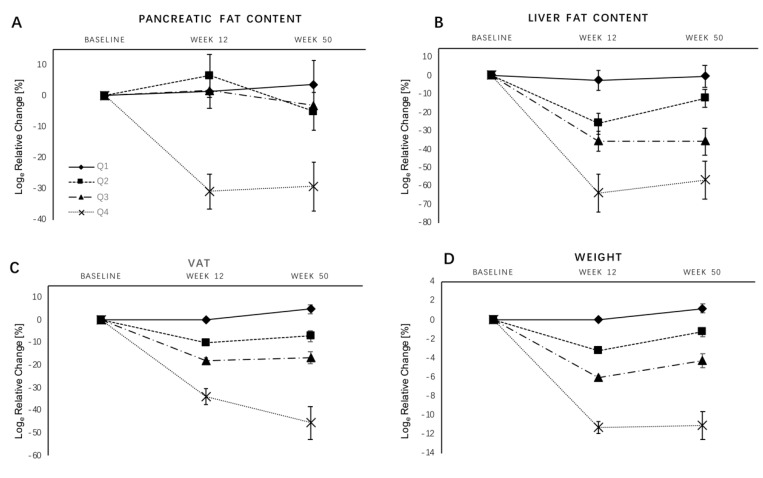
Relative changes in pancreatic fat content, liver fat content, VAT, and weight by weight-loss quartiles. (**A**) Pancreatic fat content; (**B**) liver fat content; (**C**) VAT, visceral adipose tissue; (**D**) weight. Data are shown as mean ± SEM for Log_e_ relative change with baseline values as the reference.

Relative changes in weight, VAT, PFC, and LFC between baseline and week 12 and baseline and week 50 across weight-loss quartiles are shown in Table 3 and Figure 2. Relative changes in weight between baseline and week 12 across the quartiles Q1 to Q4 were 0.0 ± 0.2%, −3.2 ± 0.1%, −6.1 ± 0.2%, and −11.3 ± 0.6%, respectively. Changes between baseline and week 50 were 1.2 ± 0.5%, −1.3 ± 0.5%, −4.3 ± 0.7%, and −11.1 ± 1.5%, respectively.

Relative changes in PFC between baseline and week 12 were 1.3 ± 6.7%, 6.5 ± 6.9%, 1.8 ± 5.4%, and −30.8 ± 5.7%, respectively, for Q1 to Q4, and 3.6 ± 5.9%, −5.1 ± 6.1%, −3.0 ± 7.9%, and −29.2 ± 7.8%, respectively, for Q1 to Q4 between baseline and week 50. There was a significant difference for changes in PFC across all weight-loss quartiles (*p* < 0.01) and for the comparison between Q1 vs. Q4 (*p* = 0.01), while there were no differences (*p* > 0.05) for Q1 vs. Q2 and Q1 vs. Q3. The relative decrease in pancreas fat content in Q4 across the intervention phase (−30.8 ± 5.7%) and follow-up phase (−29.2 ± 7.8%) was significantly greater compared to Q1, Q2, and Q3 (week 12: 1.3 ± 6.7%, 6.5 ± 6.9%, 1.8 ± 5.4%; week 50: 3.6 ± 5.9, −5.1 ± 6.1, −3.0 ± 7.9, respectively).

Upon further adjustment for height-standardized VAT, the differences in the changes in PFC between Q1 and Q4 were no longer statistically significant (*p* = 0.07, baseline–week 12; *p* = 0.37, baseline–week 50) (see Table 3).

## 4. Discussion

The present study focused on changes in PFC upon moderate weight loss in overweight and obese non-diabetic participants over a 12-week dietary intervention and 38-week follow-up phase. We showed significant decreases in PFC with weight loss among individuals in the highest weight-loss quartile. However, this difference did not remain statistically significant when adjusting for changes in VAT. Unlike VAT, which correlated with several metabolic biomarkers (insulin, triglycerides, and CRP), there were no such correlations with respect to PFC. Overall, these results suggest that decreases in VAT, rather than PFC, are crucial for metabolic improvements upon moderate weight loss in overweight and obese non-diabetics.

The clinical significance of PFC is currently under debate. Previous studies revealed strong correlations between PFC, BMI, LFC, SAT, and VAT, the latter of which is in line with our results [11,15]. Heni et al. [23] found that pancreatic fat is negatively associated with insulin secretion in participants with impaired fasting glucose (IFG) and/or impaired glucose tolerance (IGT), but not in individuals with normal glucose tolerance (NGT). Our study did not separate individuals into NGT, IGT, and IFG as our study participants were non-diabetic, metabolically rather healthy overweight or obese individuals. Our results were in line with Van der Zijl et al. [24], who demonstrated that pancreatic fat had no direct relationship with beta-cell function in individuals with impaired glucose metabolism. Moreover, Kühn et al. [6] also found no association between pancreatic steatosis and glycemic status. Our analysis has revealed no relevant association between PFC and fasting glucose metabolism biomarkers (glucose, insulin, homeostatic model assessment for insulin resistance (HOMA-IR), HbA1c). Tene et al. [11] discovered the same finding in 277 healthy obese participants. It has been proposed that dynamic glucose changes may be limited to diabetic participants [13]. Furthermore, as our glycemic measurements only indicate fasting and not postprandial glucose, correlations between pancreatic fat and beta-cell function cannot be excluded.

Our study also found no correlations between PFC and lipid biomarkers, while Wong et al. [25] found that hypertriglyceridemia was independently associated with a fatty pancreas. However, Rossi et al. [26] discovered that a decrease in pancreas fat content was not associated with lipid biomarkers decrement in a weight loss trial, which is in line with our results. Thus, the evidence on associations between PFC and functional parameters remains heterogeneous.

In contrast to PFC, our results suggest that LFC correlates with lipid biomarkers (triglycerides, HDL) and glucose metabolism biomarkers (glucose, insulin, HbA1c, HOMA-IR), while VAT correlates with insulin and triglycerides, but also CRP and leptin. A possible explanation could be the important role of liver fat content and VAT in glucose and lipid metabolism [27]. Thus, insulin resistance may be related to LFC and VAT but not pancreatic fat content among overweight and obese non-diabetics.

In contrast to PFC, VAT, which has been recognized as a major risk factor of metabolic and cardiovascular diseases [28], was associated with several metabolic biomarkers in our cohort. In agreement with our findings, Campos et al. [29] found, in a study of 172 obese adolescents after long-term weight-loss therapy, that the reduction of visceral fat was an independent predictor of insulin resistance with positive correlations with total cholesterol (TC), low-density lipoprotein (LDL), TGs, glucose, insulin, HOMA-IR, and hepatic enzymes. The positive association between VAT and PFC has been reported earlier [23,30], but not in all studies [24,31]. Rossi et al. found that VAT was the main predictor of pancreatic fat deposition in a smaller study [30]. It is interesting that apart from VAT, other potential associated predictors, including LFC, BMI, and waist circumference, showed only weaker correlations with PFC. This finding is consistent with Heber et al. [15], who stated that VAT was the only independent predictor of pancreatic fat. After applying a linear mixed model adjusted for sex, age, and VAT loss, our results showed that the PFC reduction was not independent from VAT loss. In contrast, a previous study by Tene et al. [11] found that PFC loss was independent from VAT loss. Similarly, Van der Zijl et al. [24] observed no significant association between VAT and steatotic pancreas. Covarrubias et al. [32] also discovered no relationship between PFC change and VAT change, while VAT was not a predictor of PFC reduction. One explanation for these conflicting findings might be the use of different methods for measuring pancreatic fat content in these studies. Unlike some previous studies that used a single volume of interest to measure pancreatic fat steatosis with MR spectroscopy [24], our study used multiple ROIs, considering the heterogeneous distribution of pancreatic fat tissue, especially in the pancreatic body and tail regions, which are more likely infiltrated by adipose tissue [33]. Our study used MRI-derived PDFF for PFC measurement. The MRI-derived multi-echo GRE technique with PDFF is a highly established method that provides quantitative information of fat deposition in different organs, including the liver, pancreas, kidney, and vertebral body [34]. The liver is the most published organ for PDFF, and liver PDFF is a highly reliable and accurate non-invasive method proven against biopsy and MR spectroscopy [35,36].

The association between weight loss and reduction in PFC was not linear in our study, and only weight loss >7.5% (Q4) resulted in a significant loss of pancreatic fat. However, in contrast to decreases in PFC, decreases in LFC and VAT with weight loss were linear in our study. Steven et al. [13] found that pancreatic fat content did not change in individuals with normal glucose tolerance (NGT) before and after bariatric surgery, despite a comparable mean decrease of 12.8% in body weight. Vogt et al. [14] also discovered, in a low-calorie weight-loss program, that weight loss leads to a reduction in visceral fat and liver fat, while pancreatic fat remains unchanged in obese diabetic participants. Unlike our study, both of these studies included a small number of participants and shorter intervention periods [13,14]. In agreement with our results, Gaborit et al. [12] found a 43.8% PFC loss six months after bariatric surgery, induced by weight loss of 24.6%. Rossi et al. [26] observed a 42.3% reduction in pancreatic lipid content after a three- to six-month intervention phase with 8.9% weight loss. Differences in the imaging techniques used for PFC measurement as well as differences in the study population could be responsible for these inconsistent findings.

Overall, our study and others suggest that decreases in VAT, LFC, and PFC with weight loss may not be proportional. In our study group, changes in LFC were greater than those in VAT and PFC, which is in line with a study by Rossi et al. [26], in which the same amount of weight loss by dietary restriction lead to greater reductions in LFC than in PFC. Pinnick et al. [37] observed that pancreatic fat could be stored in adipocytes among pancreatic cells in addition to lipid drops in pancreatic cells, while liver fat is located inside hepatic cells. Our findings could partially be explained by the observation that weight loss may lead to a more rapid decrease in triglycerides inside pancreatic and hepatic cells than inside adipocytes between pancreatic cells [26].

The following limitations of our study should be considered. First, the study population only included healthy overweight and obese participants. Individuals with diabetes and pre-diabetes were not included, and we did not perform an oral glucose tolerance test (OGTT). Thus, our findings may not apply to patients with (pre-)diabetes, and PFC may have more clinical relevance among these patients than among metabolically healthy overweight and obese individuals. For this project, the original study cohort consisting of three study groups was re-classified upon post-hoc analysis into four quartiles based on weight loss for the purpose of analyzing changes in PFC. Second, the analysis of pancreatic fat content relied on MR-derived estimates as due to possible complications, the performance of biopsies in healthy volunteers was not possible. However, our method of measurement using MRI-derived PDFF maps is evaluated has previously been evaluated in the literature [15].

In conclusion, this study shows a decrease in pancreatic fat content if weight loss exceeds 7.5%. The reduction in PFC is not independent from VAT loss and shows no association with lipid, glycemic, or inflammatory biomarkers.

## Figures and Tables

**Table 1:** Characteristics of the four weight-loss groups (Q1 to Q4) at baseline (T0), *n* = 137.

	Q1	Q2	Q3	Q4
	≤2%	>2% to ≤4.5%	>4.5% to ≤7.5%	>7.5%
	*n =* 35	*n =* 34	*n =* 35	*n =* 33
Women [*n*(%)]	20 (57)	15 (44)	19 (54)	18 (55)
Age [year]	51.0 ± 6.3	51.2 ± 8.3	51.2 ± 7.8	47.4 ± 8.3
Weight [kg]	94.3 ± 15.8	94.0 ± 14.0	93.3 ± 15.5	95.0 ± 14.2
Height [cm]	171.2 ± 10.5	173.8 ± 9.8	173.4 ± 10.7	173.3 ± 7.9
BMI [kg/m^2^]	32.1 ± 4.1	31.1 ± 3.7	30.9 ± 3.4	31.6 ± 3.7
Waist circumference [cm]	105.6 ± 12.3	105.5 ± 10.7	102.4 ± 11.4	103.9 ± 11.8
Systolic blood pressure (mmHg)	139.6 ± 11.0	132.2 ± 14.0	136.6 ± 14.4	140.0 ± 21.9
Diastolic blood pressure (mmHg)	90.1 ± 8.1	86.0 ± 81	87.3 ± 7.7	86.9 ± 9.8
Metabolic syndrome [*n*(%)]	9 (25.7)	3 (8.8)	5 (14.3)	5 (15.2)
**Fat depositions**				
VAT [L]	5.3 ± 2.2	5.0 ± 2.2	4.8 ± 2.0	4.7 ± 2.0
SAT [L]	13.1 ± 4.6	11.2 ± 2.8	12.1 ± 3.9	12.9 ± 4.0
Pancreas fat content [%]	10.3 ± 5.1	7.7 ± 4.1	10.1 ± 11.2	8.6 ± 4.2
Liver fat content [%]	7.1 ± 4.4	8.8 ± 7.8	7.9 ± 6.5	7.4 ± 4.9
**Glucose metabolism**				
Glucose [mg/dL]	93.4 ± 8.0	93.2 ± 6.8	94.8 ± 6.8	91.8 ± 8.0
Insulin [mU/L]	14.7 ± 7.8	12.6 ± 7.4	10.8 ± 5.1	11.2 ± 5.4
HbA1c [%]	5.5 ± 0.4	5.5 ± 0.3	5.5 ± 0.3	5.4 ± 0.3
HOMA-IR	3.4 ± 1.9	3.0 ± 1.8	2.6 ± 1.2	2.6 ± 1.3
IGF-1 [ng/mL]	114.6 ± 34.3	124.3 ± 34.1	111.3 ± 33.3	105.1 ± 27.0
**Liver function tests**				
ALT [U/L]	25.1 ± 7.2	31.5 ± 14.1	26.9 ± 12.1	24.4 ± 9.7
AST [U/L]	21.8 ± 4.0	25.8 ± 6.9	22.5 ± 4.0	22.3 ± 5.0
GGT [U/L]	29.7 ± 13.9	26.3 ± 16.1	30.4 ± 19.8	24.3 ± 12.1
**Lipid metabolism**				
Triglycerides [mg/dL]	139.4 ± 64.9	136.1 ± 89.3	143.9 ± 93.2	108.3 ± 53.5
Cholesterol [mg/dL]	211.4 ± 34.1	202.1 ± 35.9	214.4 ± 36.0	203.3 ± 34.5
HDL [mg/dL]	54.0 ± 15.0	52.6 ± 14.3	56.8 ± 13.7	52.9 ± 14.9
LDL [mg/dL]	129.5 ± 26.0	120.7 ± 25.0	128.8 ± 26.5	128.7 ± 29.5
**Inflammation**				
CRP [mg/pL]	7.0 ± 8.6	4.3 ± 5.4	3.7 ± 2.8	3.8 ± 3.8
IFNγ [ng/μL]	16.6 ± 15.8	16.7 ± 24.8	17.2 ± 16.6	11.6 ± 8.9
TNFα [ng/μL]	4.2 ± 2.7	4.4 ± 2.8	4.9 ± 2.7	4.1 ± 2.5
IL-6 [ng/μL]	2.0 ± 1.7	1.8 ± 3.4	1.3 ± 0.8	1.3 ± 1.1
IL-8 [ng/μL]	10.7 ± 4.4	14.0 ± 23.0	10.0 ± 4.7	10.6 ± 5.2
**Adipokines**				
Adiponectin [ng/mL]	15.7 ± 8.3	18.1 ± 11.4	17.0 ± 11.2	19.4 ± 13.2
Leptin [ng/mL]	29.7 ± 25.1	20.4 ± 19.9	21.2 ± 15.1	29.4 ± 29.0
Resistin [ng/mL]	5.6 ± 2.5	5.5 ± 2.0	5.3 ± 1.5	6.4 ± 3.3

Data are shown as mean ± SD. Abbreviations: ALT, alanine transaminase; AST, aspartame transaminase; BMI, body mass index; CRP, C-reactive protein; GGT, gamma-glutamyl transpeptidase; HDL, high-density lipoprotein; HOMA-IR: homeostatic model assessment for insulin resistance; IFNγ, interferon gamma; IGF-1, insulin-like growth factor 1; IL-6, interleukin 6; IL-8, interleukin 8; LDL, low-density lipoprotein; SAT, subcutaneous adipose tissue; TNFα, tumor necrosis factor-α; VAT, visceral adipose tissue.

**Table 2:** Spearman’s correlations between anthropometric parameters, blood pressure, metabolic parameters, and pancreatic fat content, liver fat content, and VAT.

	Pancreatic Fat Content		VAT		Liver Fat Content	
	rho	*p*-Value	rho	*p*-Value	rho	*p*-Value
BMI	0.269	0.002 *	0.600	<0.001 *	0.364	<0.001 *
Weight	0.292	0.001 *	0.667	<0.001 *	0.399	<0.001 *
Waist circumference	0.250	0.004 *	0.671	<0.001 *	0.477	<0.001 *
Systolic blood pressure (mmHg)	0.053	0.760	0.223	0.009	0.151	0.080
Diastolic blood pressure (mmHg)	0.027	0.535	0.324	<0.001 *	0.208	0.015
**Fat depositions**						
VAT	0.411	<0.001 *	1.000		0.586	<0.001 *
SAT	0.261	0.003 *	0.536	<0.001 *	0.360	<0.001 *
Pancreatic fat content	1.000		0.411	<0.001 *	0.195	0.026
Liver fat content	0.195	0.026	0.586	<0.001 *	1.000	
**Glucose metabolism**						
Glucose	0.086	0.328	0.260	0.003 *	0.255	0.003 *
Insulin	0.081	0.361	0.481	<0.001 *	0.454	<0.001 *
HbA1c	0.039	0.658	0.269	0.002 *	0.332	<0.001 *
HOMA-IR	0.084	0.340	0.482	<0.001 *	0.463	<0.001 *
IGF-1β	−0.040	0.655	−0.023	0.799	−0.039	0.658
**Liver function tests**						
ALT	0.152	0.084	0.317	<0.001 *	0.548	<0.001 *
AST	0.076	0.389	−0.033	0.706	0.197	0.025
GGT	0.060	0.500	0.257	0.003 *	0.221	0.011
**Lipid metabolism**						
Triglycerides	0.102	0.250	0.381	<0.001 *	0.337	<0.001 *
Cholesterol	−0.033	0.706	0.137	0.120	0.099	0.262
HDL	−0.178	0.042	−0.370	<0.001 *	−0.278	0.001 *
LDL	−0.004	0.960	0.168	0.055	0.087	0.324
**Inflammation**						
CRP	0.151	0.086	0.384	<0.001 *	0.145	0.100
IFNγ	0.015	0.870	0.006	0.943	−0.002	0.980
TNFα	−0.008	0.932	0.019	0.830	−0.003	0.972
IL-6	0.030	0.736	0.197	0.025	0.118	0.180
IL-8	0.024	0.790	0.064	0.468	0.077	0.381
**Adipokines**						
Adiponectin	0.067	0.450	−0.015	0.866	−0.036	0.683
Leptin	0.062	0.482	0.344	<0.001 *	0.165	0.060
Resistin	0.080	0.366	0.111	0.211	−0.017	0.850

* Correlation is significant at the 0.01 level (two tailed). Abbreviations: ALT, alanine transaminase; AST, aspartame transaminase; BMI, body mass index; CRP, C-reactive protein; GGT gamma-glutamyl transpeptidase; HDL, high-density lipoprotein; HOMO-IR: homeostatic model assessment for insulin resistance; IFNγ interferon gamma; IGF-1, insulin-like growth factor 1; IL-6, interleukin 6; IL-8, interleukin 8; LDL, low-density lipoprotein; SAT, subcutaneous adipose tissue; TNFα, tumor necrosis factor-α; VAT, visceral adipose tissue.

**Table 3:** Mean values and relative change (%) in weight, visceral adipose tissue, pancreatic fat content, and liver fat content in week 12 and week 50 across the weight-loss quartiles.

		Baseline	Week 12	Log_e_ relative Change (Baseline–Week 12)	*p*-Value	Week 50	Log_e_ Relative Change (Baseline–Week 50)	*p*-Value
		Mean ± SD	Mean ± SD	Mean ± SEM		Mean ± SD	Mean ± SEM	
**Weight [kg]**	Q1	94.3 ± 15.8	94.3 ± 15.8	0.0 ± 0.2	<0.01 *	95.5 ± 16.3	1.2 ± 0.5	<0.01 *
	Q2	94.0 ± 14.0	91.0 ± 13.6	−3.2 ± 0.1		93.5 ± 13.5	−1.3 ± 0.5	
	Q3	93.3 ± 15.5	87.8 ± 14.6	−6.1 ± 0.2		89.3 ± 15.8	−4.3 ± 0.7	
	Q4	95.0 ± 14.2	84.9 ± 12.8	−11.3 ± 0.6		85.3 ± 14.0	−11.1 ± 1.5	
**VAT [l]**	Q1	5.3 ± 2.2	5.2 ± 2.1	−0.1 ± 0.9	<0.01 *	5.6 ± 2.3	4.9 ± 2.0	<0.01 *
	Q2	5.0 ± 2.2	4.5 ± 2.0	−10.0 ± 1.3		4.8 ± 2.2	−7.1 ± 2.5	
	Q3	4.8 ± 2.0	4.1 ± 1.8	−18.1 ± 1.3		4.1 ± 1.9	−16.7 ± 2.6	
	Q4	4.7 ± 2.0	3.4 ± 1.6	−33.9 ± 3.4		3.2 ± 1.9	−45.4 ± 7.3	
**Pancreatic fat content [%]**	Q1	10.3 ± 5.1	10.6 ± 5.1	1.3 ± 6.7	<0.01 * (0.07 ^a^)	10.1 ± 4.0	3.6 ± 5.9	0.01 * (0.37 ^b^)
	Q2	7.7 ± 4.1	9.1 ± 6.1	6.5 ± 6.9		8.5 ± 6.7	−5.1 ± 6.1	
	Q3	10.1 ± 11.2	10.3 ± 12.2	1.8 ± 5.4		9.7 ± 11.4	−3.0 ± 7.9	
	Q4	8.6 ± 4.2	6.8 ± 4.3	−30.8 ± 5.7		6.5 ± 3.0	−29.2 ± 7.8	
**Liver fat content [%]**	Q1	7.1 ± 4.4	6.7 ± 4.1	−2.6 ± 5.4	<0.01 *	7.9 ± 5.8	−0.3 ± 6.1	<0.01 *
	Q2	8.8 ± 7.8	7.2 ± 6.2	−25.9 ± 5.7		8.3 ± 7.3	−12.3 ± 4.8	
	Q3	7.9 ± 6.5	5.3 ± 4.2	−35.8 ± 5.5		5.7 ± 5.6	−35.9 ± 7.3	
	Q4	7.4 ± 4.9	3.5 ± 1.7	−63.9 ± 10.3		3.6 ± 1.9	−56.9 ± 10.5	

Data from 137 participants were included; statistical analyses were performed among four weight-loss quartiles. Mean ± SEM of week 12 and week 50 Log_e_ relative change with baseline values as reference. *p*-values across the four weight loss-quartiles were calculated with linear mixed models (baseline to week 12, baseline to week 50) adjusted for age and sex. Abbreviations: VAT, visceral adipose tissue. ^a^ P ∆PFC (adjusted for sex, age, height standard, and ∆VAT, baseline–week 12). ^b^ P ∆PFC (adjusted for sex, age, height standard, and ∆VAT, baseline–week 50). Significant (*p* < 0.05) time-by-group interactions, with baseline values as the reference are depicted with an asterisk (*).
